# Evaluating the effects of curcumin nano-chitosan on miR-221 and miR-222 expression and Wnt/β-catenin pathways in MCF-7, MDA-MB-231 and SKBR3 cell lines

**DOI:** 10.1186/s13000-024-01468-3

**Published:** 2024-02-16

**Authors:** Touba Eslaminejad, Seyed Noureddin Nematollahi-Mahani, Marzieh Lotfian Sargazi, Mehdi Ansari, Vida Mirzaie

**Affiliations:** 1 Department of Anatomy, Afzalipour School of Medicine, Kerman University of Medical Sciences, Kerman, Iran; 2 Physiology Research Center, Institute of Neuropharmacology, Kerman University of Medical Sciences, Kerman, Iran; 3 Departments of Drug and Food Control, Faculty of Pharmacy, Kerman University of Medical Sciences, Kerman, Iran

**Keywords:** Breast cancer, micro RNAs, miR-221, miR-222, Wnt/β-catenin

## Abstract

**Background:**

Breast cancer is one of the most common diseases worldwide that affects women of reproductive age. *miR-221* and *miR-222* are two highly homogeneous microRNAs that play pivotal roles in many cellular processes and regulate the Wnt/β-catenin signaling pathway. Curcumin (CUR), a yellow polyphenolic compound, targets numerous signaling pathways relevant to cancer therapy. The main aim of this study was to compare the ability of chitosan curcumin nanoparticle (CC-CUR) formulation with the curcumin in modulating *miR-221* and *miR-222* expression through Wnt/β-catenin signaling pathway in MCF-7, MDA-MB-231 and SK-BR-3 breast cancer cell lines.

**Method:**

Chitosan-cyclodextrin-tripolyphosphate containing curcumin nanoparticles (CC-CUR) were prepared. Cytotoxicity of the CUR and CC-CUR was evaluated. Experimental groups including CC-CUR, CUR and negative control were designed. The expression of *miR-221* and *miR-222* and Wnt/β-catenin pathway genes was measured.

**Results:**

The level of *miR-221* and *miR-222* and *β-catenin* genes decreased in MCF-7 and MDA-MB-231 cells and *WIF1* gene increased in all cells in CC-CUR group. However, the results in SK-BR-3 cell line were unexpected; since miRs and *WIF1* gene expressions were increased following CC-CUR administration and *β-catenin* decreased by administration of CUR.

**Conclusion:**

Although the composite form of curcumin decreased the expression of *miR-221* and *miR-222* in MCF-7 and MDA cells, with significant decreasing of *β-catenin* and increasing of *WIF1* gene in almost all three cell lines, we can conclude than this formulation exerts its effect mainly through the Wnt/β-catenin pathway. These preliminary findings may pave the way for the use of curcumin nanoparticles in the treatment of some known cancers.

## Introduction

Breast cancer is the most common malignancy in women and the second leading cause of cancer-related deaths worldwide. Global statistics show an increase in the incidence of breast cancer mostly in developing countries [1]. It has pathophysiological appearance and its high prevalence and differences in response patterns to various treatment modalities as well as differences in clinical outcomes provide a strong rationale for identifying natural and synthetic treatment agents [2]. Breast cancer is divided into 3 types based on the presence or absence of different proteins in the cancerous cells. 70% of breast cancer cases are **hormone receptor–positive** type which has either estrogen receptor (ER) or progesterone receptor (PR) protein in the cancer cells; 15–20% of cases are **ERBB2-positive** (formerly known as human epidermal growth factor receptor 2 (HER2)-positive) which has high levels of ERBB2 protein on the cancer cells; and the remaining 15% of cases are **triple-negative** (TNBC) type that does not have ER, PR, or ERBB2 protein in the cancer cells [3, 4]. Among these types of breast cancers, TNBC is the most aggressive tumor with poor prognosis in young women. Because estrogen and progesterone receptors are not expressed in this type, TNBC do not benefit from hormone therapy and although they respond to chemotherapies, prognosis remains poor [5, 6]. The lack of appropriate treatment has prompted researchers to identify the molecular pathways involved in the proliferation and growth of these tumors, which can be a new hope for TNBC tumor treatment [7, 8]. Micro RNAs are a group of small non-coding RNAs with a length of about 18–24 nucleotides. These molecules control gene expression by inhibiting the translation of target mRNAs [9]. Studies have shown that Mic RNAs regulate the expression of a large number of genes involved in various biological processes e.g., proliferation, differentiation, survival, migration, apoptosis, and cell death. Mic RNAs are classified into oncogenic and tumor suppressor groups [9]. The *miR-221* and *miR-222* are two highly homogeneous mic RNAs that act as a gene cluster (*miR-221/222*) in cellular regulation [10]. The expression levels of *miR-221* and *miR-222* varies in different types of breast cancers [11, 12]. *MiR-221/222* regulate the Wnt/β-catenin signaling pathway, which plays an important role in fetal growth, proliferation, polarity, and cell migration [13]. Wnt protein contains a large family of signaling molecules that affect a large number of biological processes and cell growth. Under normal conditions, the *β-catenin* gene is rapidly phosphorylated by the action of a multiprotein compound, marked and decomposed by ubiquitination [4]. *miR-221/222* are positive regulators at different levels of *β-catenin* gene expression. *β-catenin* is a key regulator in the Wnt signaling cascade and appears to be involved in the transcription of genes involved in estrogen-independent growth in cancer cells [13]. Wnt-inhibitory factor-1 (*WIF1*) is a secreted protein in Wnt/β-catenin signaling pathway that binds to Wnt proteins and inhibits their activities [4].

Medicinal plants have long been used to treat various diseases including cancers [14], but the way to deliver the drug to these cells and as a result the effectiveness of the drug is one of the important factors that should be taken into consideration. Curcumin (diferuloylmethane), an orange-yellow polyphenolic compound of the curcuminoid family is a natural product isolated from the rhizome of Curcuma longa. For centuries, it has been used in medicinal preparations and also as a food colorant. In recent years, extensive in vitro and in vivo studies have suggested that curcumin possesses activity against cancer, viral infection, arthritis, amyloid aggregation, oxidation and inflammation [15]. Curcumin exerts anticancer effects primarily by activating apoptotic pathways in cancer cells and inhibiting pro-cancer processes, including inflammation, angiogenesis and metastasis [16]. Curcumin targets numerous signaling pathways relevant to cancer therapy, including those mediated by p53, Ras, phosphatidylinositol-3-kinase, protein kinase B, Wnt/β-catenin and the mammalian target of rapamycin [15]. At the molecular level, curcumin increased Tp53 gene expression [17, 18]. Curcumin may inhibit the ability of camptothecin, mechlorethamine and doxorubicin to induce apoptosis by up to 70% in MCF-7, MDA-MB-231 and BT-474 human breast cancer cells [15]. On the other hand, its bioavailability is low, due to its low solubility [19]. Therefore, drug delivery systems based on nanoparticles can increase its stability and solubility in water [20]. There are several ways to formulate curcumin, including nanocrystals, micelles, and other conjugates that increase its permeability. Chitosan is a cellulose-dependent mucopolysaccharide obtained by deactivating chitin. Chitosan is a non-toxic, biodegradable, biocompatible and safe molecule. Chitosan nanoparticles showed a better physicochemical, antibacterial, and biological properties than the other vectors due to their small size and surface-to-volume ratio [21, 22]. Therefore, the aim of this study was to formulate the curcumin in chitosan nanoparticles and then evaluation and comparing the effect of parent curcumin and its nano form (composite) on the *miR-221* and *miR-222* and Wnt/β-catenin signaling pathway in three different kinds of breast cancer cells, MCF-7, MDA-MB-231 and SK-BR-3.

## Materials and methods

Chitosan (low molecular weight, viscosity 20–300 cP, 1% wt in 1% acetic acid), sodium tripolyphosphate (STPP), Dulbecco’s modified eagle’s medium (DMEM), penicillin–streptomycin (100 μg/ml), phosphate-buffered saline (PBS), 3-(4,5-Dimethylthiazol-2-yl)-2,5-diphenyltetrazolium bromide (MTT reagent), Tween 80 and Span 80 were all purchased from Sigma-Aldrich (Sigma-Aldrich company. Mo, USA). β- Cyclodextrin (CD) with molecular weight *of 1135* g/mol and curcumin were obtained from Merck Company (Darmstadt, Germany).

### Manufacture of chitosan-cyclodextrin-tripolyphosphate containing curcumin nanoparticles (CS/CD/TPP/CUR) (CC-CUR)

Nanoparticles were prepared by ionotropic gelling. To achieve a homogeneous solution of curcumin, first, a solution of tween in water and curcumin in span was made, and then the two solutions were mixed and homogenized well by ultrasonication. Chitosan noncomplex was prepared according to our previous study [21]. Briefly, Chitosan aqueous solution (CS) was mixed with a cyclodextrin (CD) aqueous solution (with the cross linker TPP) using a magnetic stirrer. In order to enter the curcumin into the nanoparticle, 1 mg/ml of curcumin solution was directly placed in tripolyphosphate and added to the chitosan mixture and stirred to allow the complete formation of the system for 30 min. The final product was used for further experiments.

### Evaluation of physicochemical characteristics of produced nanoparticles

The particle size and zeta potential of CC-CUR nanoparticles were performed using Zetasizer (Malvern Instrument, USA) device. Fourier-transform infrared spectroscopy (FT-IR) was used to identify chemical properties of CC-CUR. Scanning electron microscope (SEM) was used to measure CC-CUR nanoparticles’ size, morphology and topography. X-Ray diffraction analysis (XRD) was used to determine the crystallographic structure of CC-CUR nanoparticles. Energy dispersive X-ray (EDX) microanalysis was used to identify the elemental composition of CC-CUR nanoparticles. Differential scanning calorimetry (DSC) as a thermo-analytical technique was used to determine the thermal properties of CC-CUR by using a differential scanning calorimeter.

### Curcumin release profile

Phosphate-buffered saline (PBS, pH 5.8) was used to evaluate the release of curcumin from nanoparticles at different time intervals. After dialyzing the samples by dialysis membrane (MWCO 12,000 Da) against PBS, free curcumin was quantified in the supernatant by Optizen 3220UV (South Korea) instrument at 430 nm absorbance.

### Cell culture and cytotoxicity evaluation

Cytotoxicity of CC-CUR and CUR was assessed by MTT assay according to the manufacturer’s protocol. MCF-7 cells were seeded in 96-well plates with a density of 1 × 10^4^ cells/well in three replicates and incubated for 24 h in a 37 ^○^C humidified incubator with 5% CO2 in air. Twenty μl of different concentrations (0.05, 0.5 and 5 mg/ml) of CUR and CC-CUR were mixed with 80 μl serum free medium and kept at room temperature for 20 min. The mixtures were transferred into each well containing different cells and incubated for 48 h. 10 μl of MTT solution was added to each well and incubated for four hours. The supernatant was replaced by 100 μl of DMSO. The absorbance was read at 570–630 nm by an ELISA reader (Bio-tech Instruments, USA).

### Transfection of cells

DMEM-F12 supplemented with 10% FBS was used and the cells were maintained in a humidified incubator with 5% CO_2_ at 37 °C. One day before transfection, cells at a density of 4˟10^5^ were seeded in the fresh serum-free medium (2% FBS), in 6 cm dishes until they reached 60–70% confluence. After 24 h of incubation, the medium was changed with 100 μl of CUR and CC-CUR nanocomposite in serum-free medium without antibiotics. RNA extraction was done according to a published protocol [23].

### RNA extraction and gene expression

Cancerous cells were lysed by adding 1 ml of RiboEx™ (Cat No., 301–001, GeneAll, Portugal) reagent following the manufacturer’s instructions. Phenol/chloroform was used to remove proteins to obtain an A_260_:A_280_ ratio of 1.81 ± 0.06, using a UV spectrophotometer (Thermo Scientific™ Nanodrop 2000). cDNA was synthesized using the Parstous cDNA synthesis kit (Cat No., A101161, Parstous Biotechnology company, Mashhad, Iran) according to the manufacturer’s recommendations. 0.1 μg of total RNA was used for every first strand cDNA synthesis as follow: primer annealing at 25 ºC for 10 min, denaturation at 47 ºC for 60 min and heat inactivation at 85 ºC for 5 min.

### Real time PCR

Real-time PCR was performed using the Sina SYBR blue reaction mix without low ROX (Sina Clone) in a magnetic induction cycler (mic) Real-time PCR system, using 1.5 μl of each cDNA sample, and 10 pmol of each primer. The reaction program was 95 ºC for 7 min, followed by 40 cycles of 95 ºC for 15s, 65 ºC for 20s, and 72 ºC for 35s. The C_T_ (threshold cycle) values were analyzed using 2^−∆∆CT^ methods. *β-actin* and *GAPDH* were used as the endogenous reference genes for normalizing the fold change in gene expression. The intrinsic expression of the control group was normalized by the reference genes and set to one. The sequence of primers is shown Table 1.

**Table 1:** Primer sequence of genes

Genes	Primers sequences
*miR 221*	F: GGTCTGGGGCATGAACCT
	R: GAGAACATGTTTCCAGGTAGCC
*miR 222*	F: GCTGCTGGAAGGTGTAGGTA
	R: GATGCCATCAGAGACCCAGTA
*β-catenin*	F: TGGAGGAGAGACAGCCCTTA
	R: ATTGTCCACGCTGGATTTTC
*WIF1*	F: TGTTTCAGAGGGGAAAATGG
	R: GGACATTGACGGTTGGATCT
*GAPDH*	F: GAGCCACATCGCTCAGACAC
	R: CATGTAGTTGAGGTCAATGAAGG
*β-actin*	F: AGCACAGAGCCTCGCCTTT
	R: CACGATGGAGGGGAAGAC

### Statistical analysis

All experiments were performed in triplicate and in three independent assays. Data were expressed as mean ± SD. Microsoft Excel 2019 was used to manage data and draw graphs. Data were statistically analyzed by using SPSS software version 16. *p* < 0.05 was considered statistically significant.

## Results

### Evaluation of physicochemical characteristics of nanoparticles

**Fig. 1:**
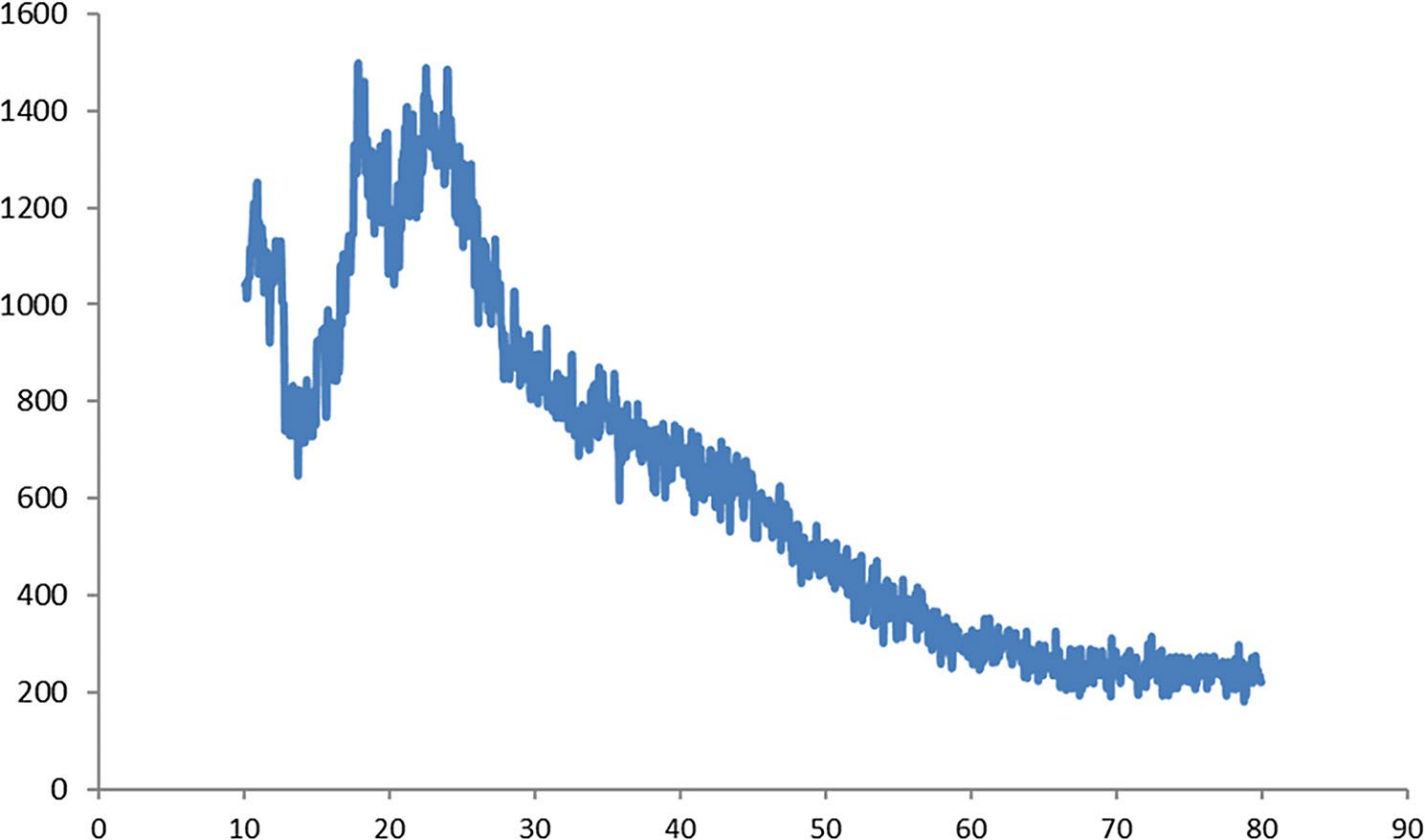
XRD pattern of CS/CD/TPP nano-complex

The X-ray diffraction patterns taken for chitosan nano-complex are presented in Fig. 1. Chitosan exhibited a crystalline peak at 2θ = 20.20°, with a slight shift to a higher diffraction angle, indicating better crystalline nature of the chitosan complex. The lower intensity exhibited by the diffraction peaks of CS/CD/TPP nano-complex revealed that they are amorphous in nature. The absence of other diffraction peaks related to impurities in the XRD patterns of CS/CD/TPP nano-complex confirmed their high purity. The ionic interaction between TPP and –NH^3+^ of chitosan molecules has resulted in the formation of CS/CD/TPP nano-complex. In case of CS/CD/TPP nano-complex, the intensity of diffraction peaks was increased as a consequence of transforming amorphous chitosan into crystallized form after reaction with TPP. The diffraction peak of pure chitosan which was usually observed at 20.20° has slightly shifted to a lower value (19.85°), this can be attributed to the reaction of CS/CD/TPP nano-complex with TPP and the crystallized structure of CS/CD/TPP nano-complex.

**Fig. 2:**
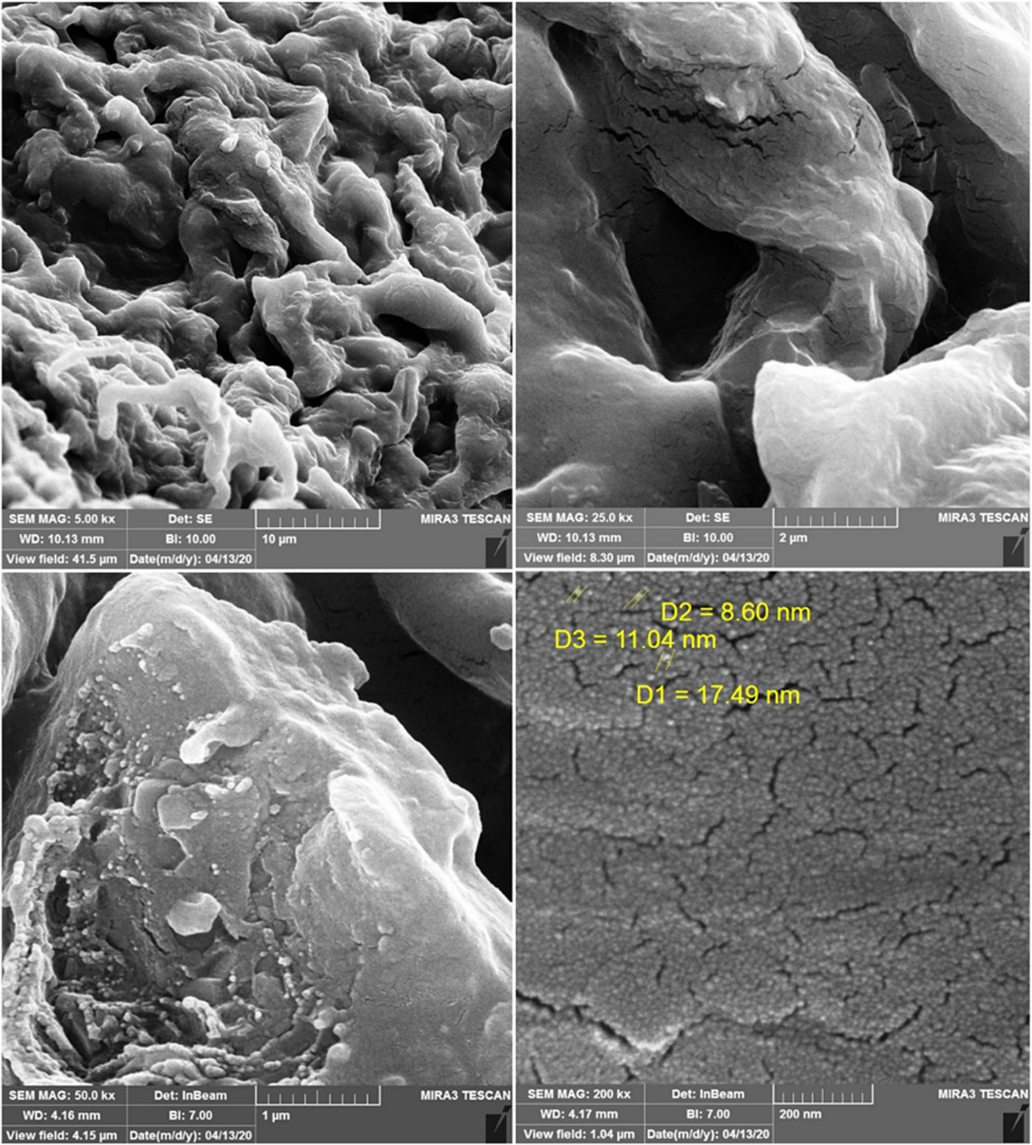
SEM images of CS/CD/TPP nano-complex exhibit almost sphere-like shape with smooth, regular surface, and less aggregation shape

The SEM images of CS/CD/TPP nano-complex samples are shown in Fig. 2. The SEM image of CS/CD/TPP nano-complex sample showed presence of diverse shapes of particles with sizes from 50 to 150 nm. In addition, its lower magnification image revealed that chitosan contained irregular sheet-like particles. Moreover, the surface of the chitosan particles was smooth in the absence of voids.

**Fig. 3:**
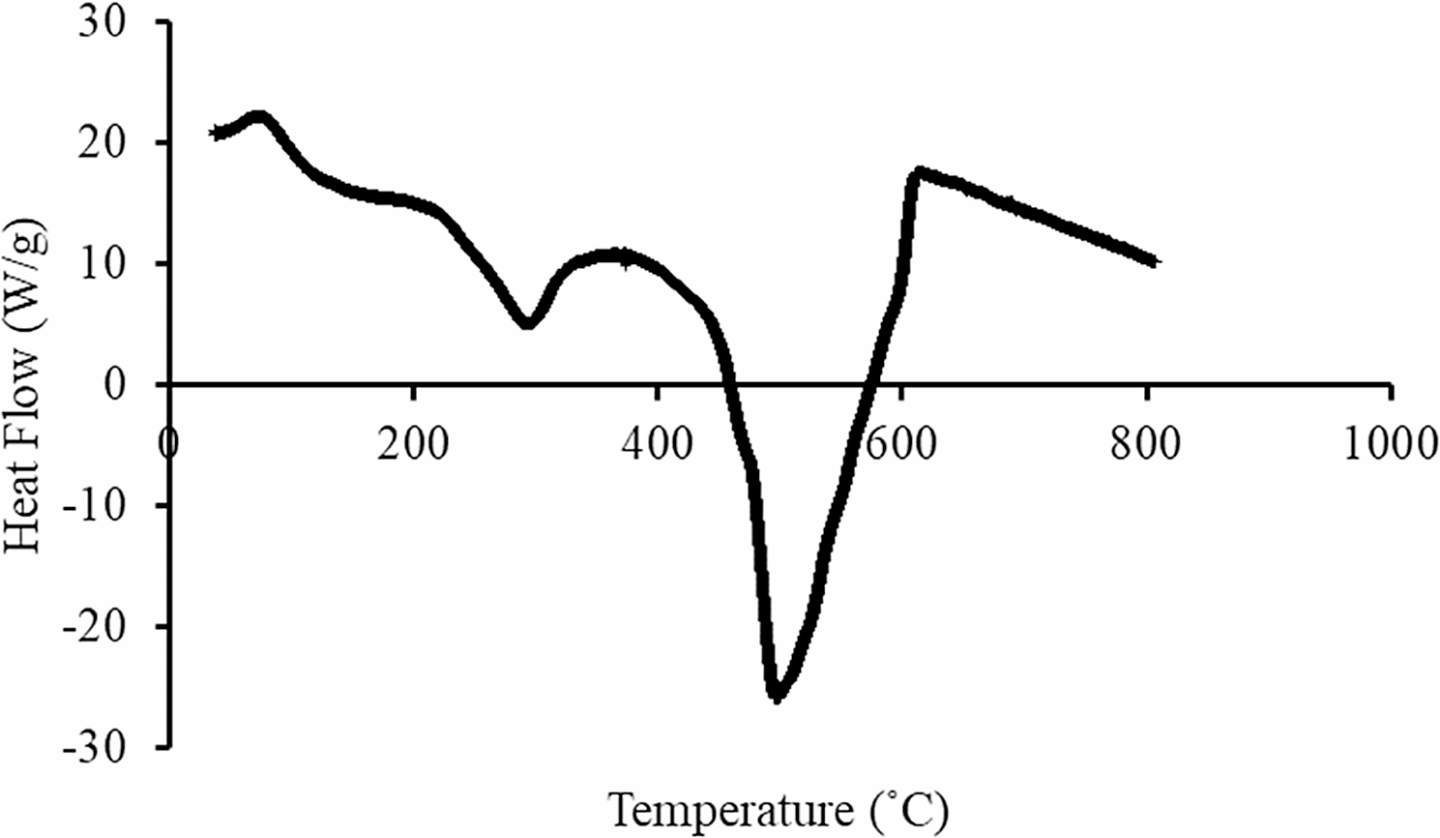
DSC analysis of CS/CD/TPP nano-complex was performed at a temperature between 0 and 800 °C

DSC curve of CS/CD/TPP nano-complex showed both endothermic and exothermic peaks. An endothermic peak at 100 °C is probably due to water loss from the hydrophilic groups; whilst an exothermic peak at 306.0 °C indicates degradation due to dehydration and de polymerization (Fig. 3).

### Curcumin release profile

**Fig. 4:**
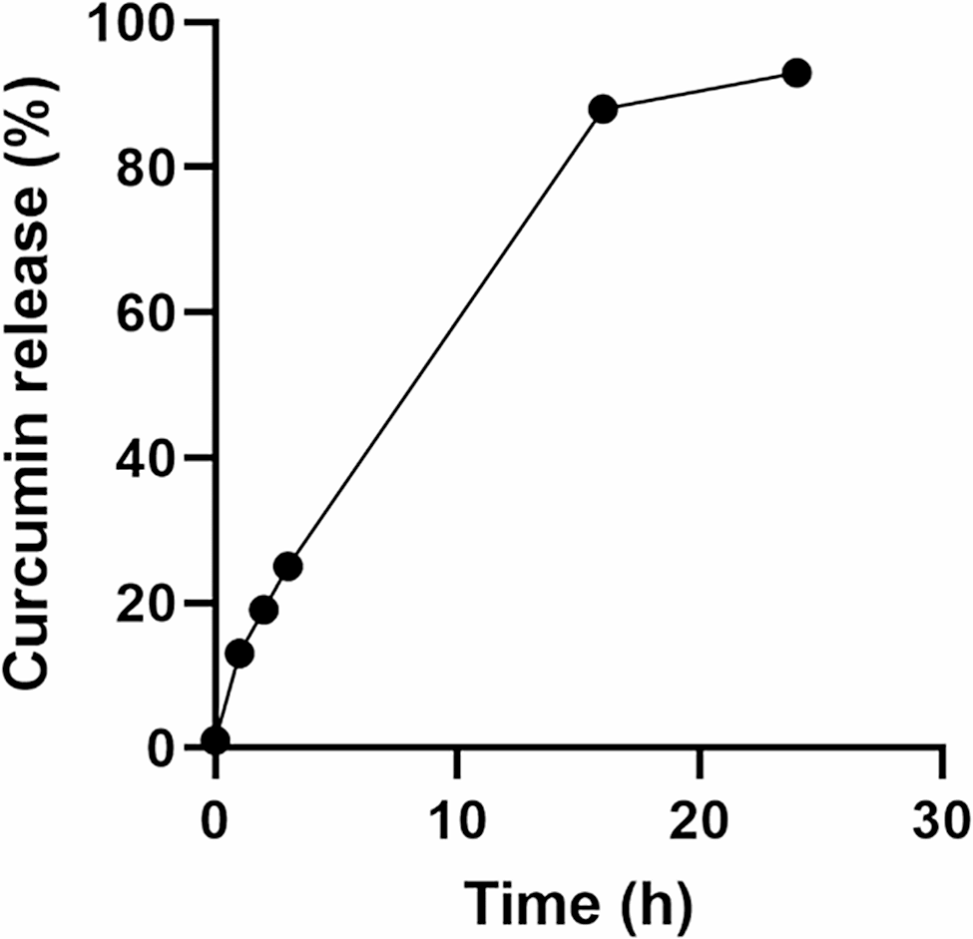
Release profile of curcumin from CS/CD/TPP nano-complex through dialysis membrane into PBS (pH 5.8) as receiver medium

Data of the release pattern of curcumin showed that 0, 13, 19, 25, 88 and 93% of the curcumin was released in 0, 1, 2, 3, 16 and 24 h, respectively (Fig. 4).

### Cytotoxicity

**Fig. 5:**
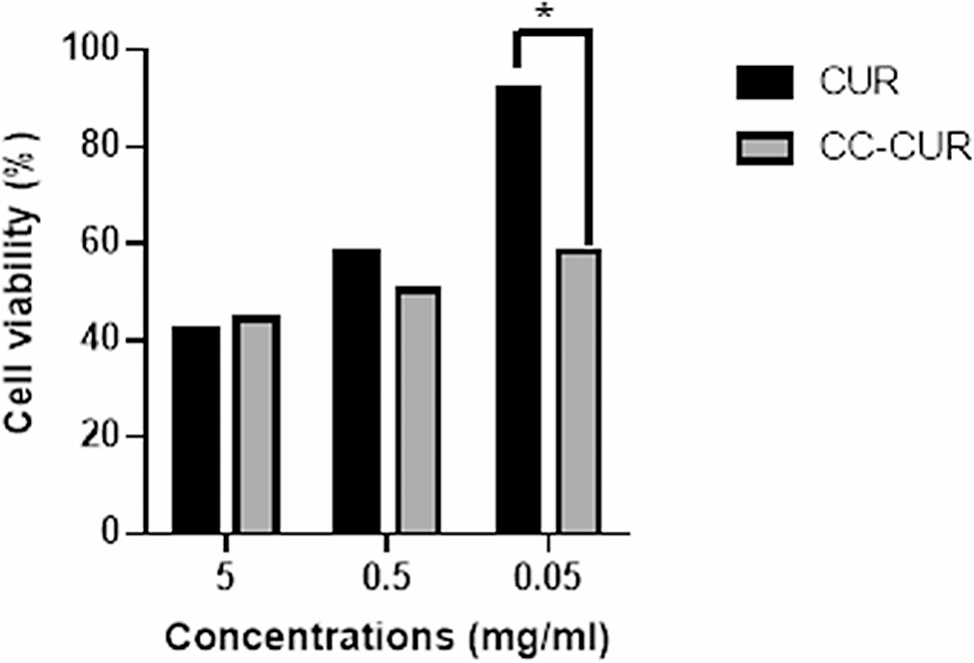
Cell viability profile of CUR and CC-CUR (*p ≤ 0.05)

MCF7 cells were treated in different concentrations for 48 h and the viability of the cells were measured by MTT assay. Results showed that difference between the cell viability of CUR and CC-CUR groups was much clearer at concentrations of 0.5 and 0.05 mg/ml (*p* < 0.05). It should be noted that CC-CUR nanocomposite was more toxic than CUR at lower concentrations (0.05 mg/ml) (Fig. 5).

### Gene expression analysis

**Fig. 6:**
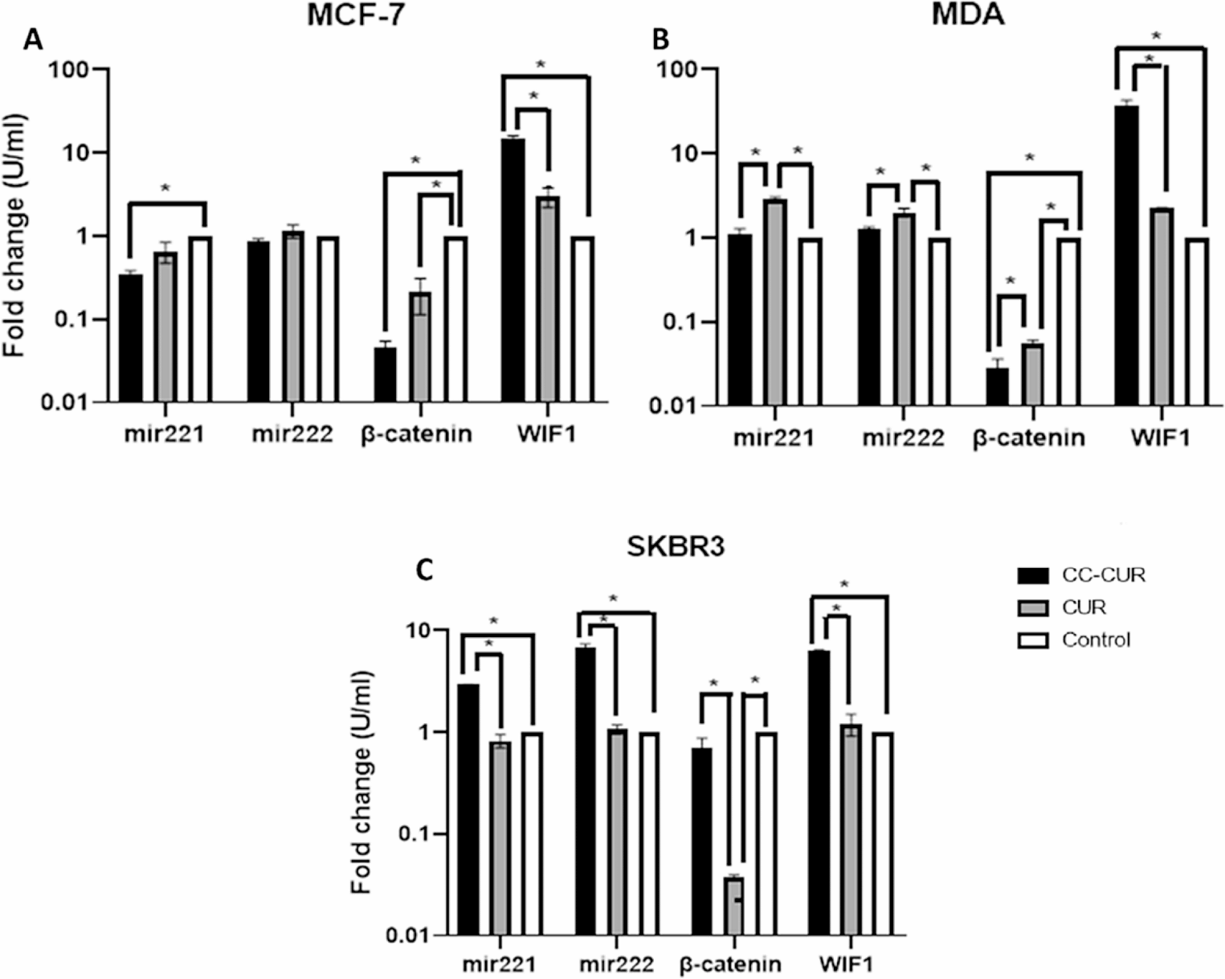
*mir221*, *mir222*, *β-catenin* and *WIF1* gene expression level in (**A**); MCF-7, (**B**); MDA and (**C**); SKBR3 cells

Comparing the expression of different genes in MCF-7 cells indicated that the level of *miR-221* gene in the CC-CUR group was significantly decreased (*P* < 0.01). CUR also decreased the expression level of this gene, although it did not reach a significant level. *MiR-222* level did not show any significant difference between groups although its level in CC-CUR group was lower than in the other groups. Expression of *β-catenin* gene decreased significantly in CC-CUR and CUR groups in comparison to the control group, although this decrease was higher in the CC-CUR group (*P* < 0.0001). The level of *WIF1* gene expression in the CC-CUR group was significantly increased compared to both CUR and control groups (*P* < 0.0001). CUR also non-significantly increased the level of *WIF1* gene expression in comparison to the control group (Fig. 6A).

In MDA-MB-231 cells the level of *miR-221* and *miR-222* genes expression in CUR group was significantly higher than in the control and CC-CUR groups while there was no significant difference between the CC-CUR and control groups. *β-catenin* level significantly decreased in both CC-CUR and CUR groups in comparison to the control group (*P* < 0.0001), a significant difference between CC-CUR and CUR groups was also detected. The level of *WIF1* gene significantly increased in CC-CUR group compared to the control and CUR groups (*P* value < 0.001). *WIF1* expression level in the CUR group also non-significantly increased compared to the control group (Fig. 6B).

The level of *miR-221* and *miR-222* genes in SKBR3 cells significantly increased in CC-CUR group compared to the CUR and control groups (*P* < 0.0001), However, the expression level of these genes in CUR group was almost similar to the control group. CUR significantly decreased the expression level of *β-catenin* gene compared to both CC-CUR and control groups. *WIF1* expression level in the CC-CUR group was significantly higher than that in the CUR and control groups (*P* < 0.0001) (Fig. 6C).

## Discussion

*miR-221* and *miR-222* are two oncomiRs that are consistently up-regulated in breast cancer tissues [24]. Activation of *β-catenin* by *miR-221* and *miR-222* promotes estrogen-independent growth of cancer cells, whereas Wnt-inhibitory factor-1 (*WIF1*) is suppressed by these miRNAs [25]. *WIF1* acts as a tumor suppressor gene in Wnt/β-catenin signaling pathway [26]. Curcumin inhibits breast cancer cell proliferation by altering expression of signaling proteins, including Ras, phosphatidylinositol-3-kinase (PI3K), protein kinase B (Akt), mammalian target of rapamycin (mTOR) and Wnt/β-catenin pathway [15]. Therefore, targeting oncogenes and tumor suppressor genes is one of the most effective methods in the treatment of cancers. The present study investigated the possible anti-cancer effects of nano curcumin (CC-CUR) against different types of breast cancer cell lines by analyzing the expression level of *miR-221*, *miR-222* and *β-catenin* as oncogene and *WIF1* as a tumor suppressor gene. The results of cell viability assay which showed more toxicity of CC-CUR nanocomposite at lower concentrations than CUR reveals that in lower doses, CC-CUR has higher toxicity than CUR on breast cancer cells and to achieve more toxicity, higher doses of parent curcumin is needed which can be explained by the fact that the nanocurcumin is more soluble in addition to higher cellular uptake.

MCF-7 cell line is a Luminal A subtype expressing estrogen receptor (ER) and progesterone receptor (PR). This cell line is considered non-invasive and non-tumorigenic in vivo unless supplemented with estrogen, meaning they rely on estrogens for growth [27]. MDA-MB-231 is a basal type hormone-independent TNBC that is thought to be more aggressive and invasive than other breast cancer subtypes [28]. Also, SKBR3 cells represent basal-like BC that are estrogen-independent (her2^+^, ER/PR^−^) [29].

The results of previous studies showed that the expression of *miR-221* and *miR-222* was increased in breast cancer tissues compared with normal breast tissues [10, 30]. In addition, the level of *miR-221* and *miR-222* were associated with the advanced clinical stage and different tumor types [31]. *miR-221* and *miR-222* represses multiple negative regulators of the Wnt/β-catenin signaling pathway, including *WIF1*, *SFRP2*, *DKK2*, and *AXIN2*, to activate the pathway. Notably, the patient survival is inversely correlated with *miR-221* and *miR-222* expression level whereas it is positively correlated with that of *WIF1*, *DKK2*, *SFRP2*, and *AXIN2* genes [13].

Our results indicated that not only the level of *miR-221* and *miR-222* genes are different in different types of intact breast cancers, but also the response rate of these genes to treatment is different. In our study the expression level of *miR-221* and *miR-222* reached to the lowest level in MCF7 cells compared to MDA-MB-231 and SKBR3 cells after treating with curcumin. Therefore, we can conclude that the effects of curcumin on the expression of *miR-221* and *miR-222* is more prominent in hormone dependent cancerous cells and it may lead to the hypothesis that these cells are more sensitive with better responding to certain treatments.

Many researches indicated that curcumin could acts as an anti-cancer agent in breast cancer cell line due to the regulation of different pathways such as *miR-221* and *miR-222* and Wnt/β-catenin [32, 33]. In this study we observed different effects of curcumin on different cell lines. Evaluating the effects of parent curcumin (CUR) and its nanocomposite form (CC-CUR) on three different cells lines showed that CC-CUR down-regulated the expression of *miR-221* and *miR-222* in MCF-7 cells, although its effect on the Wnt/β-catenin pathway was more prominent. CUR also modulated these pathways but the effect of the CC-CUR was more significant.

Liu et al., reported that Wnt/β-catenin signaling is hyperactivated in TNBC, promoting the invasive potential of TNBC. Inhibiting *miR-221/222* expression in a TNBC cell line (MDA-MB-231) suppressed its proliferation, viability, epithelial-to-mesenchymal transition, and migration. Whereas expression of *miR-221/222* in a non-TNBC line (MCF-7) promotes all the aforementioned cancer characteristics [13]. However, the effects of our treatment on Wnt/β-catenin pathway on both MDA-MB-231 and MCF-7 cells was similar and CC-CUR effect was significantly better than CUR. While the expression of *miR-221* and *miR-222* was not satisfactory in MDA-MB-231 cells, especially in the CUR group, as the level of these genes increased compared to other groups. The CC-CUR also could not decrease the expression level of miR genes in MDA-MB-231 cells and its level was equal to the control group.

The results in SKBR3 cells were quite unexpected as the expression level of miR genes in the CC-CUR group increased considerably. Treatment of SKBR3 cells with CC-CUR significantly increased the level of *WIF1* gene expression without decreasing the level of *β-catenin*. On the other hand, CUR did not change the level of miR genes compared to the control group, while it significantly decreased *β-catenin* expression. Kim et al. claimed that the expression levels of *miR-221* and *miR-222* were associated with the aggressive characteristics of breast cancer subtypes and their expression level vary in breast cancers according to tumor characteristics [34]. Generally, the gene expression values in our study indicated that curcumin in its parent or composite forms affects cancerous cell death through the Wnt/β-catenin pathway and the significant point is that this effect is not basically by modulating the expression of *miR-221* and *miR-222* genes and shows the effect of other paths involved in this process.

*WIF1* has been shown to be down-regulated in various human cancers, including breast cancer, and has been regarded as a tumor suppressor gene [26]. Rubin et al. demonstrated that loss of *WIF1* could trigger Wnt/β-catenin signaling and thereby contributes to tumor invasion and metastasis [35]. Therefore, up-regulation of this gene can be a promising strategy in controlling tumor proliferation and metastasis and our results indicated that in all three cell lines the level of *WIF1* significantly increased after using CC-CUR.

Previous studies demonstrated that up-regulated *miR-221/222* may simultaneously suppress multiple inhibitors of Wnt/β-catenin signaling pathway such as *WIF1*, supporting the idea that miRNA represents a potent activator of the pathway [13]. While our results indicating the importance of other genes in activation of Wnt/β-catenin pathways. Therefore, it remains to be clarified whether *miR-221* and *miR-222* regulate other signaling pathways to promote oncogenesis or other genes regulating Wnt/β-catenin pathway.

## Conclusion

We conclude that since different genes are involved in cancers, investigating different signaling pathways contributed in these diseases is very important. MCF-7 cells are more sensitive to curcumin treatment especially its nanocomposite form than MDA-MB-231 and SKBR3 cells due to the expression of *miR-221* and *miR-222* genes. Furthermore, curcumin decoration with chitosan nanoparticles strongly affected Wnt/β-catenin pathway by reducing *β-catenin* and increasing *WIF1* gene in almost all three breast cancer cell lines, the findings that need to be further investigated in different cancers as well as in vivo conditions.

## Data Availability

No datasets were generated or analysed during the current study.
